# Effects of Medical Male Circumcision (MC) on Plasma HIV Viral Load in HIV+ HAART Naïve Men; Rakai, Uganda

**DOI:** 10.1371/journal.pone.0110382

**Published:** 2014-11-21

**Authors:** Godfrey Kigozi, Richard Musoke, Nehemiah Kighoma, Stephen Watya, David Serwadda, Fred Nalugoda, Noah Kiwanuka, Fred Wabwire-Mangen, Aaron Tobian, Fredrick Makumbi, Ronald Moses Galiwango, Nelson Sewankambo, James Nkale, Grace Kigozi Nalwoga, Margaret Anyokorit, Tom Lutalo, Ronald Henry Gray, Maria Joan Wawer

**Affiliations:** 1 Rakai Health Sciences Program, Entebbe, Uganda; 2 Urocare clinic, Kampala, Uganda; 3 School of Public Health, Makerere University College of Health Sciences, Kampala, Uganda; 4 College of Health Sciences, Makerere University, Kampala, Uganda; 5 Department of Epidemiology, Johns Hopkins University, Bloomberg School of Public Health, Baltimore, Maryland, United States of America; Tulane University, United States of America

## Abstract

**Background:**

Medical male circumcision (MC) of HIV-infected men may increase plasma HIV viral load and place female partners at risk of infection. We assessed the effect of MC on plasma HIV viral load in HIV-infected men in Rakai, Uganda.

**Methods:**

195 consenting HIV-positive, HAART naïve men aged 12 and above provided blood for plasma HIV viral load testing before surgery and weekly for six weeks and at 2 and 3 months post surgery. Data were also collected on baseline social demographic characteristics and CD4 counts. Change in log_10_ plasma viral load between baseline and follow-up visits was estimated using paired t tests and multivariate generalized estimating equation (GEE).

**Results:**

Of the 195 men, 129 had a CD4 count ≧350 and 66 had CD4 <350 cells/mm^3^. Men with CD4 counts <350 had higher baseline mean log_10_ plasma viral load than those with CD4 counts ≧350 cells/mm^3^ (4.715 vs 4.217 cps/mL, respectively, p = 0.0005). Compared to baseline, there was no statistically significant increase in post-MC HIV plasma viral loads irrespective of CD4. Multivariate analysis showed that higher baseline log_10_ plasma viral load was significantly associated with reduction in mean log_10_ plasma viral load following MC (coef.  = −0.134, p<0.001).

**Conclusion:**

We observed no increase in plasma HIV viral load following MC in HIV-infected, HAART naïve men.

## Introduction

Three trials of male circumcision (MC) show that MC reduces male HIV acquisition by 50–60% [1]–[3]. WHO/UNAIDS recommended that, although MC should not be promoted for HIV-infected men, they should not be denied the service if they request it for reasons other than HIV prevention and have no medical contraindications to surgery [4]. Benefits of medical male circumcision to HIV positive men include prevention of genital ulcer disease; prevention of sexually transmitted infections such as HSV2, HPV to self and sexual partner; better genital hygiene; minimizes stigma etc. However, a trial of MC in HIV-infected men with CD4 counts >350 cells/mm^3^ to assess effects on HIV transmission to women partners suggested that HIV transmission may be higher following MC in couples who initiated sexual intercourse before wound healing was complete [5]. This study also found an increase in male plasma HIV viral load four weeks after MC^5^, and it was speculated that the increased viremia may be due to surgical stress and temporary immune-suppression. An increase in plasma viral load following MC could lead to increased risk of HIV transmission to HIV- negative female partners [6]–[7]. A recent study conducted in Kenya showed no significant increase in viral load after MC [8].

To determine whether MC of HIV+ men affected plasma HIV viral load, we assessed the effect of MC surgery on plasma HIV viral load during the immediate post- MC period among HAART naive HIV-infected men with CD4+ T cell counts <350 and ≧350 cells/mm^3^.

## Methods

### Ethics statement

The study was reviewed and approved by the Higher Degrees, Research and Ethics Committee (HDREC) of the Makerere University, School of Public Health (MUSPH), by the Scientific and Ethics Committee (SEC) of the Ugandan Virus Research Institute (UVRI), by Western Institutional review Board (WIRB) in the US, and by the Uganda National Council of Science and Technology (UNCST).

We conducted a prospective cohort study in Rakai district, Uganda between 2009 and 2011. All uncircumcised HIV-infected men aged 12 and above who requested free MC services and had no contraindication to surgery were invited to participate in the study and asked to provide written informed assent if minor or consent if adult. Parents or guardians provided written informed consent for minors aged less than 18 years. All HIV-infected men were referred for HIV care. Referral notes were given to clients to take to an HIV care clinic of their choice for further counseling and consideration for HAART.

All HIV-infected men who consented to participate in the study (n = 332) were enrolled. A random sample of HIV-negative men who came for the free MC service were concurrently enrolled in a parallel study of MC wound healing to avoid stigmatization of the HIV-positive participants.

Men were offered free individual voluntary counseling and testing (VCT), though acceptance of VCT was not a prerequisite for free MC. On the day of surgery men were provided with education on HIV prevention and MC through group sessions. Information was provided on risks and benefits of MC, on the surgical procedure, wound care and the need to abstain from intercourse until complete wound healing was certified. Men were then clinically assessed for contraindications to surgery by trained medical or clinical officers. Participants without contraindications were asked to consent to participation in the study.

Data were collected at baseline and follow-up visits by trained male interviewers using structured questionnaires. Information collected included socio-demographic, health and behavioral characteristics, symptoms of surgery related complications, resumption of sex, and condom use. Blood for HIV testing, plasma viral load and CD4 count determination was collected prior to surgery. 96% of the surgeries were conducted by trained clinical officers and 4% by medical officers using the dorsal slit method as described in the WHO Manual for Male Circumcision Under Local Anaesthesia [9] under aseptic conditions. Postoperative instructions were given on proper wound care and use of analgesics that were provided.

All participants were followed weekly for six weeks and then at 2 and 3 months post-surgery. At these visits, venous blood was collected for plasma viral load determination, and data collected on surgery related moderate or severe adverse events and wound healing status.

HIV status was determined by two enzyme immunoassays (EIAs); Vironostika HIV-1/2 Plus O (Organon Teknika, Charlotte, NC, USA) and Murex HIV-1.2.0 (Murex Biotech Limited, Dartford, UK), which were run in series. Samples were first tested using Murex Biotech EIA assay which is more sensitive, and then by the Vironostika HIV-1/2 test which is more specific. Samples discordant on the two EIA tests and those that were in the gray zone on Vironostika HIV-1/2 were subjected to Western blot (WB) confirmation (HIV-1 Western Blot; Bio-Merieux-Vitek) or by PCR in cases where WB result were indeterminate. Determination of CD4+ T cell counts used a three-color FACSCaliber (Becton Dickenson, New Jersey, USA). Plasma HIV-1 RNA viral loads were determined by a reverse-transcriptase–polymerase-chain-reaction (RT-PCR) assay (AMPLICOR HIV-1 MONITOR version 1.5 Roche Molecular Systems, Branchburg, N.J.). All tests were done at the Rakai Health Sciences Laboratory in Kalisizo.

### Statistical analysis

Men who were on Highly active antiretroviral therapy (HAART) were excluded from this analysis because therapy suppresses viremia which could obscure the effects of MC. Participants' characteristics were assessed at baseline stratified by CD4 counts ≧350 cells/mm^3^ and below 350 cells/mm^3^. Baseline behavioral and social demographic characteristics were compared using Chi-square tests.

Plasma HIV Viral load was log_10_ transformed. Equality of means of log_10_ plasma viral load for the two CD4 groups and by HIV care status was tested using two sample t-test.

Mean change in log_10_ plasma viral load and 95% confidence intervals were estimated by follow up visit and plotted to examine change in plasma viral load relative to the pre-MC enrollment levels. Within-individual changes in plasma viral load relative to pre-surgical levels were assessed using paired t test. Population-averaged (marginal) multivariate regression models with generalized estimating equations (GEE) estimates of robust variance were used to estimate adjusted changes in plasma viral load. A sensitivity analysis was performed in a subgroup of 111 men who had complete data on plasma viral load from week 1 to week 8 postoperatively. Analyses were performed using Stata version 12.0 (College Station, Texas).

## Results

**Figure 1:**
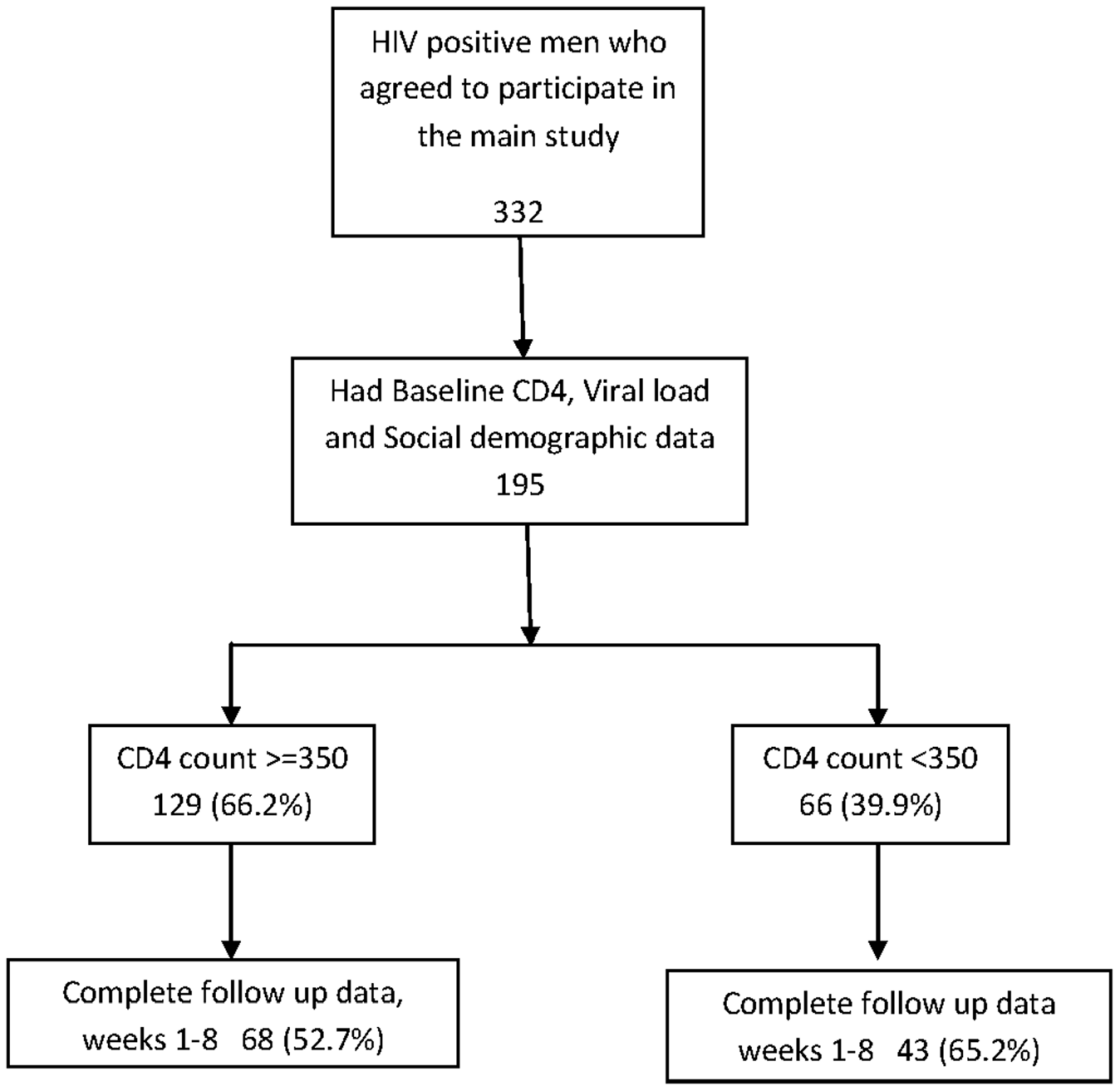
Study Flow Chart.

Figure 1 shows the study flow chart. 332 HIV-positive men agreed to participate in the main study of whom 195 had a baseline plasma HIV viral load and CD4 count, and information on social-demographic characteristics because collection of these data was initiated later in the study. Men who lacked data on any of these variables were not included in the analysis. 129 (66.2%) of the 195 men had CD4 counts ≧350 cells/mm^3^ and 66 (33.9%) had CD4 counts <350 cells/mm^3^ at baseline prior to surgery, Follow up rates were above 80% at all visits except week 6 among men with CD4≧350 cells/mm^3^ (77.5%) and week 12 for men with CD4<350 cells/mm^3^ (72.7%). Of the 111 men with plasma viral load test results at all scheduled follow-up visits from weeks 1–8, 68 had CD4≧350 cells/mm^3^ (52.7%) and 43 had CD4<350 cells/mm^3^ (65.2%)

Table 1 shows baseline behavioral characteristic by CD4 group for the 195 men included in this analysis. There were no differences in baseline characteristics. Baseline characteristics for the 111 men who had complete plasma viral load results at all follow up visits were also comparable at enrollment. We also observed no difference in baseline behavioral characteristics betwen men who were included and those who were excluded from the study.

**Table 1:** Baseline characteristics by CD4 group for the main and the sensitivity analysis sample.

	All men					Subsample with complete data at all visits 1–8				
Baseline Characteristic	HIV+ (CD4≥350)		HIV+ (CD4<350)		P-Value	HIV+ (CD4≥350)		HIV + (CD4<350)		P-Value
	No.	%	No.	%.		No.	%	No.	%	
**Total**	**129**	**100**	**66**	**100**		**68**	**100**	**43**	**100**	
**Age**										
<30	57	44.2	21	31.8	*0.120*	25	36.8	11	30.6	*0.192*
30–39	47	36.4	34	51.5		26	38.2	24	55.8	
40+	25	19.4	11	16.7		17	25.0	8	18.6	
**Marital Status**										
Married	96	74.4	49	74.2	*0.979*	51	75.0	35	81.4	*0.432*
Not married	33	25.6	17	25.8		17	25.0	8	18.6	
**Education**										
None or Primary	93	72.1	49	74.2	*0.750*	47	69.1	35	81.4	*0.151*
Secondary education or more	36	27.9	17	25.8		21	30.9	8	18.6	
**Occupation**										
Agriculture	51	39.5	24	36.4	*0.384*	32	47.1	18	41.9	*0.399*
Paid Employment	33	25.6	23	34.9		13	19.1	13	30.2	
Other forms of Employment	45	34.9	19	28.8		23	33.8	12	27.9	
**Number of sexual partners**										
0 or 1	52	40.3	20	30.3	*0.171*	30	44.1	14	32.6	*0.225*
2 or more	77	59.7	46	69.7		38	55.9	29	67.4	
**Condom use sexually active**										
No Condom use	53	41.1	29	43.9	*0.617*	25	51.0	21	58.3	*0.086*
Sometimes	36	27.9	20	30.3		15	30.6	14	38.9	
Always	13	10.1	04	6.1		9	18.4	1	2.8	
**Alcohol**										
No Alcohol use	34	26.4	18	27.3	*0.892*	20	29.4	11	25.6	*0.580*
Sometimes	87	67.4	45	68.2		44	64.7	31	72.1	
Often	08	6.2	03	4.6		4	5.9	1	2.33	
**HIV Care (Self reported)**										
HIV+ men Not in HIV Care	99	76.7	54	81.8	*0.415*	55	80.9	34	79.1	*0.815*
In care on Septrin	30	23.3	12	18.2		13	19.1	**9**	20.9	

In the 195 men, the enrollment mean log_10_ plasma viral load for men with CD4<350 cells/mm^3^ was 4.715 cps/mL which was significantly higher than for men with CD4≧350 cells/mm^3^, 4.217 cps/mL, p = 0.0005 (mean difference  = 0.498 [95% CI: 0.222, 0.774]). Similar differences were observed in the subsample with complete follow up visits (p = 0.0013).

**Figure 2:**
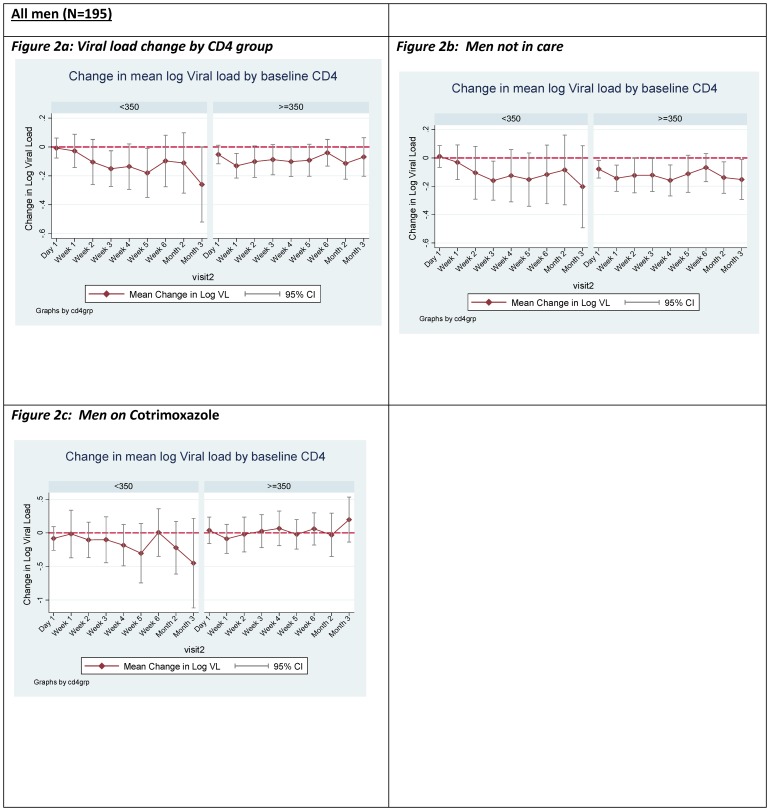
Graphs of mean differences in log_10_ viral load relative to baseline by follow-up visit.

Table 2 and figure 2 show a comparison of baseline and postoperative mean log_10_ plasma viral loads by CD4 strata. Thehe mean log_10_ plasma viral loads post-surgery were lower than the mean log_10_ plasma viral loads before surgery, and the changes of viral load were of borderline or not statistically significant at some visits (figure 2a). On further sub-analysis, among men who were in care (on Co-trimoxazole) we observed no significant change in log_10_ mean viral load in both CD4 groups (figure 2c). Among men who were not in care, we observed a borderline or non-significant decline in plasma viral load over time in both CD4 groups (figure 2b).

**Table 2:** Number, Mean log_10_ viral loads, mean difference (95%CI) and p values for change in viral load at follow up visits.

Group	Visit	Number of observations	Baseline mean log_10_ viral load (cps/mL) of men seen at each visit	Follow up visit mean log_10_ viral load(cps/mL)	Mean within individual difference in viral load (Baseline – follow up)(95%CI)	p-value
CD4> = 350	Day 1	111	4.241	4.188	0.053 (−0.012, 0.117)	0.109
	Week 1	117	4.210	4.080	0.130 (0.044, 0.216)	0.003
	Week 2	103	4.226	4.124	0.102 (−0.008, 0.211)	0.069
	Week 3	104	4.185	4.098	0.087 (−0.017, 0.192)	0.103
	Week 4	106	4.204	4.103	0.101 (−0.003, 0.205)	0.057
	Week 5	106	4.227	4.135	0.092 (−0.019, 0.203)	0.104
	Week 6	100	4.220	4.180	0.040 (−0.052, 0.132)	0.390
	Week 8	107	4.263	4.149	0.114 (0.005, 0.224)	0.041
	Week 12	109	4.206	4.137	0.069 (−0.066, 0.203)	0.314
						
CD4<350	Day 1	61	4.689	4.682	0.007 (−0.062, 0.077)	0.839
	Week 1	65	4.723	4.695	0.028 (−0.088, 0.143)	0.634
	Week 2	60	4.720	4.615	0.105 (0.052, 0.262)	0.187
	Week 3	57	4.686	4.536	0.150 (0.026, 0.274)	0.018
	Week 4	61	4.745	4.610	0.135 (−0.023, 0.293)	0.092
	Week 5	56	4.771	4.591	0.178 (0.0107, 0.349)	0.038
	Week 6	55	4.786	4.690	0.096 (−0.083, 0.275)	0.286
	Week 8	58	4.749	4.638	0.111 (−0.099, 0.320)	0.294
	Week 12	48	4.579	4.319	0.260 (−0.000, 0.520)	0.050

We observed similar trends in the subsample with complete scheduled follow up visits.

Multivariate analysis (GEE) showed that higher baseline log_10_ plasma viral load was significantly associated with reductions in mean log_10_ plasma viral load (coef.  = −0.134, p<0.001). A similar finding was observed with the subsample of men with complete follow up (p<0.001).

## Discussion

Our findings show that MC is not associated with an increase in plasma viral load post-operatively, irrespective of baseline CD4 count. Men consistently had higher mean log_10_ plasma viral loads before surgery compared to all post- surgery visits, irrespective of initial CD4, though the differences were borderline or not statistically significant.

This finding differs from our prior study of HIV+ men with CD4 counts >350 cells/mm^3^ that suggested an increase in plasma viral load at week 4 post-surgery [5]. The findings are however consistent with those from a Kenyan study that observed no change in viral load in the immediate post-circumcision period [8]. We cannot explain the differences in findings, but the current study and the recent study from Kenya suggest that postoperative increase in plasma viral load does not explain the potential increase in HIV transmission to HIV-negative women who resumed sex before the wound was completely healed [5]. We postulate that having an open surgical wound can lead to an increase in viral shedding from the open wound as was shown in the Kenyan study [8]. Viral shedding can lead to an increase in HIV transmission to HIV negative partners if circumcised men resumed sex before the wound is completely healed. In addition, the increase in viral load could potentially be explained by the amount of virus in seminal fluid. Unfortunately, this study did not assess seminal viral load.

The strengths of the current study were the frequent weekly observations and the inclusion of HIV+ men with lower CD4 counts <350. We also excluded men on HAART since the effects of therapy could mask viral load changes following MC. The findings are internally consistent. As expected, the mean log_10_ plasma viral load among men with CD4<350 cells/mm^3^ was higher than men with CD4≧350 cells/mm^3^, but enrollment CD4 count was not associated with significant changes in mean log_10_ plasma viral load postoperatively. Limitations of this study include small sample size, especially for HIV+ men with CD4 counts <350 which limited our ability to compare them to those with CD4 counts >350. Nevertheless, the study had >80% power to detect a difference or change in viral load among all HIV positive men combined (*Formula: N per group  =  (Z_α/2_+Z_β_)^2^ 2σ^2^ [1+(n−1)ρ]/nΔ^2^. Assuming: α = 0.05, Zα = 1.96, 1−β = 0.80, Z_β_ = 0.84, σ^2^ = 0.586, N = 138.* ***Where:*** *n = number of repeated measurements;* Δ *is difference in log_10_ viral load between groups being compared. (From previous Rakai studies* Δ* = 0.26 cps/mL is the change in log_10_ VL that can increase HIV transmission); p is the correlation coefficient between repeated measurements*).

Other limitations to this study include the fact that though we knew HAART status at enrollment and referred participants for care, we did not establish HAART initiation status during the 12 weeks of follow up visits. Initiation of HAART could lead to a reduction in viral load especially among men with high viral load. However, this is a short time window and would only potentially affect men with CD4<350 cells/mm^3^. Also the sample of men with CD4<350 cells/mm^3^ was small due to exclusion of men on HAART.

## Conclusion

We observed no increase in plasma HIV viral load following MC in HIV-infected men not on HAART. MC programs should continue providing services to HIV-positive men who request them and counsel them to abstain from sex until the wound is completely healed and to use condoms after they resume intercourse in order to minimize the risk of transmitting HIV to their sexual partners.
